# Endoscopic Goniosynechialysis for Acute Angle Closure Glaucoma Following Descemet’s Stripping Automated Endothelial Keratoplasty

**DOI:** 10.5005/jp-journals-10008-1250

**Published:** 2018-08-01

**Authors:** Mrinal Rana, Sunil Shah, Pravin Pandey, Imran Masood

**Affiliations:** 1 Consultant, Department of Ophthalmology, Peterborough City Hospital, North West Anglia Hospital NHS Trust, Bretton Gate, Peterborough, UK; 2 Lead Consultant, Department of Ophthalmology, Birmingham and Midland Eye Centre, UK; 3 Consultant, Department of Ophthalmology, Birmingham and Midland Eye Centre, UK; 4 Clinical Lead and Consultant, Department of Ophthalmology, Birmingham and Midland Eye Centre, UK

**Keywords:** Cohort study, Educational training, Glaucoma, Resident versus attending, Tube shunt surgery

## Abstract

We describe a new modified technique to release the peripheral iridocorneal adhesions that formed after Descemet stripping automated endothelial keratoplasty. The usual technique of goniosynechialysis was modified and performed using endoscopic fiber-optic light and camera probe to aid visualization of the adherent iris tissue and carry out uneventful 270 degrees release of adhesions. The iris tissue was gently pulled away using micro forceps. The modified technique was conceptualized, as the view from the cornea was very poor due to recent lamellar surgery and corneal oedema secondary to poorly controlled intraocular pressure. The blocked trabecular meshwork system was successfully recanalized, which allowed an adequate control of intraocular pressure. The graft survived the insult and cornea gained complete clarity giving the patient the desired vision and improved quality of life.

**How to cite this article:** Rana M, Shah S, Pandey P, Masood I. Endoscopic Goniosynechialysis for Acute Angle Closure Glaucoma Following Descemet’s Stripping Automated Endothelial Keratoplasty. J Curr Glaucoma Pract 2018;12(2):90-93.

## INTRODUCTION

Goniosynechialysis a surgical procedure used in the management of angle closure glaucoma secondary to peripheral anterior synechiae (PAS). There are limited reports in the literature on its use in the acute setting to restore trabecular function allowing aqueous drainage.^1,2^ Intraocular pressure (IOP) can rise significantly after posterior lamellar keratoplasty (PLK) involving insertion of an air bubble into the anterior chamber (AC) to aid adhesion of the donor button to the host cornea. PLK since its first description in 1956 by Tillet^3^ has undergone various modifications, however, these advances in technology have not obviated the use of air or gas in the AC to tamponade the graft to the recipient cornea. Rises in IOP have been well documented due to pupil block^4^ and iridocorneal adhesions secondary to air entrapment behind the iris.

We describe a case of acute angle closure glaucoma in a patient who underwent descemet stripping automated endothelial keratoplasty (DSAEK) and developed acute IOP rise secondary to iridocorneal adhesions and PAS. A new technique is described here using an ocular endo-scope and micro-forceps to perform goniosynechialysis, as the cornea was oedematous precluding direct visualization of the angle.

## DESCRIPTION OF CASE AND TECHNIQUE

**Fig. 1A to D:**
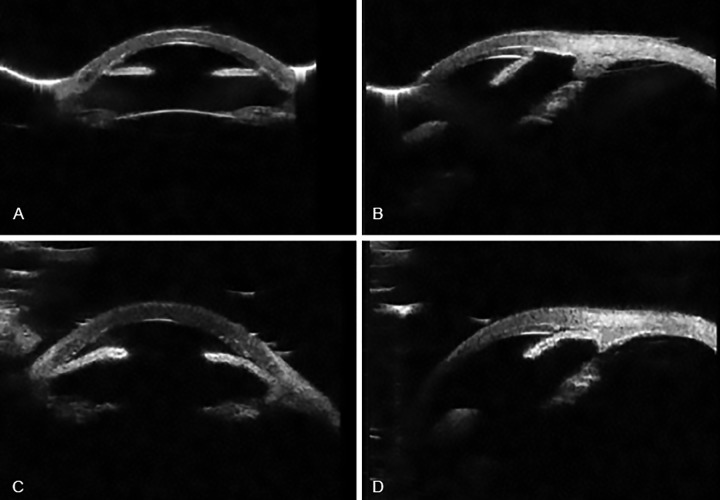
(A) and (B) Ultrasound biomicroscopy showing extensive peripheral anterior synechiae post endothelial keratoplasty causing obstruction of aqueous outflow resulting in persistent uncontrolled intraocular pressure; (C) and (D) Ultrasound biomicroscopy done after GSL shows release of peripheral anterior synechiae and an open trabecular meshwork space, which allowed adequate IOP control.

An 84-year-old Asian man known to suffer from Fuch’s endothelial dystrophy (FED) and primary open angle glaucoma underwent an uncomplicated phacoemul-sification with intraocular lens implant followed by a sequential DSAEK within 1 month post cataract surgery. The patient was on alpha agonists (Tamsulosin) for benign prostate hyperplasia. It is well known that alpha agonists cause floppy iris syndrome and this resulted in intra-operative difficulties. Due to the floppy iris, the air fill was difficult to maintain and air travelled posteriorly behind the iris causing significant anterior bowing of the iris tissue. As a full air fill of the anterior chamber is required to adequately tamponade the graft button to the host cornea, the attempt to inject air was continued. Various maneuvers were attempted to avoid air traveling behind the iris and getting trapped causing an iatrogenic iris bombe, which included the use of intracameral phen-ylephrine to dilate the pupil and also to stabilize the floppy iris. A satisfactory air fill was eventually achieved by injecting air to completely fill the anterior chamber, pushing the iris into a concave configuration and also achieving a tight globe. This full air chamber fill was maintained for 10 minutes to allow a good and effective tamponade between the host cornea and endothelial graft button allowing it to stick to the host cornea. The IOP in the immediate postoperative period was noted to 20 mm Hg. Three days later the patient presented as an emergency with an IOP of 50 mm Hg. He was treated medically with intravenous and oral acetazolamide (Diamox, slow release 250 mg, Mercury Pharmaceuticals Ltd, London, UK) and topical beta-blockers (timolol 0.5%), carbonic anhydrase inhibitors (Dorzolamide, Trusopt 2%, Merck and Co., Inc, Whitehouse Station, USA) and prostaglan-dins analogues(Latanoprost 0.005%). This reduced the IOP acutely, but the IOP remained poorly controlled over the next few weeks. The patient was referred to the glaucoma service for further management. On assessment, his vision still measured to 0.6-logMAR only, in spite of early signs of resolution of corneal oedema. Ultrasound biomicroscopy (UBM) was performed that showed circumferential iridocorneal adhesions and high PAS along the edge of the graft and in the angles (Figs 1A and B). As he was found to have extensive 360^o^ PAS, goniosynechi-alysis (GSL) was offered as the first option to reduce the IOP. A poor intra-ocular view necessitated that the GSL be performed using a new modified endoscopic technique, utilizing special micro-forceps. Immediate post-operative IOP was noted to be 14 mm Hg. This has remained stable after twelve months and has measured around 16 mm Hg on topical monotherapy (Travoprost, Travatan 0.004%, Alcon Pharmaceuticals Ltd) and persisting open angles as seen on UBM (Figs 1C and D). The graft has remained clear with a BCVA of 0.3 logMAR.

### Surgical technique

**Figs 2A to D:**
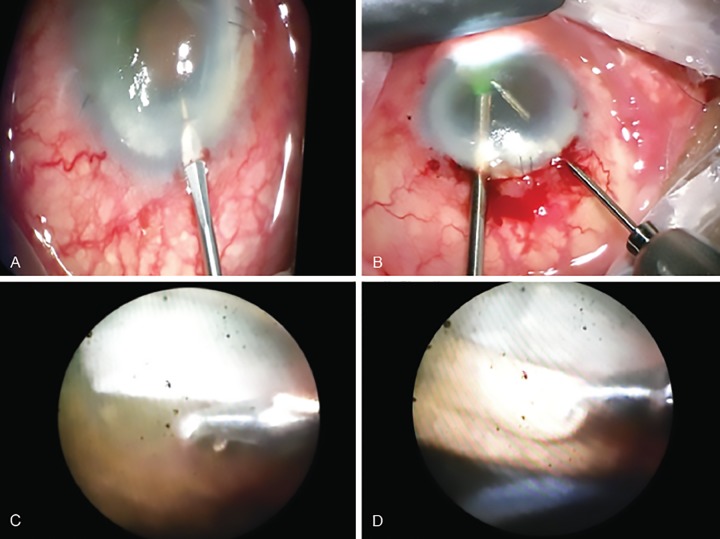
(A) MVR blade used to make 2 paracentesis incisions; (B) Endoscopic fiberoptic light probe with an attached camera and a small micro-holding forceps inserted through the paracentesis; (C) and (D) Micro-holding forceps used to grasp the anterior iris close to the synechiae and pulled to release the adhesions

The procedure was performed by IM, under general anesthesia after giving the patient intravenous mannitol 20% (200 ml over 30 min). A temporal three step corneal incision was made into the eye, taking care to avoid the graft edge (Fig. 2A). As there were iridocorneal adhesions along the edge of the graft, it was found difficult to enter the eye without disturbing the graft and lifting the edge and releasing the iridocorneal adhesions at the temporal margin. Paracenteses were also made after taking the utmost precautions at the infero-temporal and supero-temporal corneal margins. A cohesive viscoelastic substance was injected into the anterior chamber. An endoscopic fiberoptic probe was inserted from the para-centesis with a camera attached to the end of the probe (Fig. 2B). This allowed a view of the anterior chamber and iridocorneal adhesions on the monitor. Once a clear view of the angle was obtained, micro-holding forceps (MST, Redmond, WA) were introduced into the AC from the main wound. The iris was grasped peripherally with the forceps and gently pulled centripetally towards the pupil (Figs 2C and D). The process was repeated until all nasal, inferior and superior PAS and iridocorneal adhesions were released up to 270 degrees.

At the completion of the goniosynechialysis, all vis-coelastic was meticulously removed so as to avoid an IOP spike in an already compromised angle. Corneal wounds were then hydrated and sutured using 10/0 nylon (Alcon) sutures.

## DISCUSSION

Rates of glaucoma following DSAEK are difficult to ascertain owing to the shorter duration that it has been employed as a treatment modality^5^. Early results show lower rates of glaucoma, though one study done by Vajaranant et al.^6^ found that the incidence of raised IOP post DSAEK in eyes with preexisting glaucoma was up to 45%, and in eyes with no preexisting glaucoma was as high as 35%.

As a DSAEK procedure involves injecting air into the anterior chamber for a period, extension or entrapment of the air bubble over or behind the iris respectively can bring an acute rise in IOP needing urgent intervention to avoid optic nerve compromise or vascular occlusion. The mechanism of causing such acute episodes can vary according to the position of the air bubble and the iris. If the air bubble is in front of the iris and covers the pupil completely for a considerable period, the subsequent rise in IOP is due to a pupil block mechanism. If on the other hand the air gets pushed and entrapped behind the iris, it may cause peripheral anterior synechiae leading to a secondary angle closure and rise in IOP. Prevention and mitigation of such mechanisms are carried out by the use of long-acting mydriatics following completion of the procedure or post operatively to prevent pupil block^7^ and intraoperative sweeping of the iris to release the entrapped air. Other methods can also be undertaken to prevent pupil block by creating prophylactic laser peripheral iridotomies or intraoperative surgical iridotomies with the help of a 20 gauge vitrectomy cutter.

Lee et al.^7^ published their findings of secondary angle closure due to air migration behind the iris and causing anterior displacement of the iris with subsequent PAS. This occurred more commonly than pupil block and accounted for, in six of the 13 patients under study with high IOP post procedure. Only one patient in their cohort had pupil block. The effects of high IOP and PAS-led to graft failure in five out of the seven. This mechanism of air entrapment behind the iris was probably the cause of high pressure in our patient as a shallow AC was noted immediately post operatively with no clinical evidence of pupil block.

Microendoscopic trabecular surgery in the management of chronic open angle glaucoma^8^ or closed angle glaucoma^9^ has been well described to be a very effective technique, which can be performed in the presence of corneal opacification or haze that would preclude an adequate visualization of the angle and further inter-ventional therapy.^10^

We believe that this is the first description of endoscopic GSL in a post DSAEK eye, which is modified from the method that has been described in the management of synechial angle closure when performed in conjunction with phacoemulsification.^11^ In this case, the usual method of GSL would have been very difficult due to extremely poor view from a cloudy cornea, as the DSAEK procedure had been done very recently within 2 months. It would have caused more trauma, bleeding, and inflammation, hence our modification of taking the endoscopic route. Also, we prefer the use of microforceps, which appear to be less traumatic and allow for a measured amount of force to be applied. In our experience, this is a safe intervention though complications with the use of surgical GSL have been described and include fibrinous uveitis, hyphaema, and iridodialysis.^12^

As the DSAEK procedure is taking more precedence over the conventional penetrating keratoplasty due to various advantages- shorted healing times and fewer complications, we may find the incidence of this air-induced secondary glaucoma to rise in spite of various preventative measures to avoid pupil block and air entry behind the iris. This new modified technique will help glaucoma surgeons release the iridocorneal adhesions and help reduce intraocular pressure spikes or persisted elevation and reduce the risk of pressure-induced optic neuropathy and graft failure.

## CONCLUSION

### What was known

- Procedure designed to strip off goniosynechiae or peripheral anterior synechiae to open the occluded angles.
- Use of iris or cyclodialysis spatula or bent 25 gauge needle has been recommended to manually break the synechiae.
- Need for clear cornea to have a good view from a direct gonioscopic lens.

### Clinical significance

- Use of endoscopic technique allows the procedure to be performed even when the corneal clarity is an issue.
- No need for a gonioscopic lens
- Use of micro-forceps allow good grip of the iris tissue and controlled movement can be made is release of the tissue avoiding tissue damage and hyphaema.
